# Occurrence, Dominance, and Combined Use of Antibiotics in Aquaculture Ponds

**DOI:** 10.3390/toxics13100892

**Published:** 2025-10-18

**Authors:** Emmanuel Bob Samuel Simbo, Zhiyuan Ma, Longxiang Fang, Sampa Morgan, Sahr Lamin Sumana, Meshack Chubwa Maguru, Mbonyiwe Chakanga, Haggai Gondwe, Alpha Thaimu Bundu, Liping Qiu, Chao Song, Shunlong Meng

**Affiliations:** 1 Wuxi Fisheries College, Nanjing Agricultural University, Wuxi 214081, China; esimbo58jr@gmail.com (E.B.S.S.); 13908833719@163.com (Z.M.); morgansampa@yahoo.com (S.M.); meshackmaguru924@gmail.com (M.C.M.); mbonyiwechakanga@gmail.com (M.C.); gondwehaggai@yahoo.com (H.G.); thaimubundualpha@gmail.com (A.T.B.); 2 Freshwater Fisheries Research Center, Chinese Academy of Fishery Sciences, Wuxi 214081, China; fanglongxiang@ffrc.cn (L.F.); qiulp@ffrc.cn (L.Q.); 3 Laboratory of Quality & Safety Risk Assessment for Aquatic Products on Environmental Factors (Wuxi), Ministry of Agriculture and Rural Affairs, Wuxi 214081, China; 4 Key Laboratory of Control of Quality and Safety for Aquatic Products, Ministry of Agriculture and Rural Affairs, Beijing 100141, China; 5 Key Laboratory of Freshwater Fisheries and Germplasm Resources Utilization, Ministry of Agriculture and Rural Affairs, Wuxi 214081, China; 6 College of Oceanography and Ecological Science, Shanghai Ocean University, Shanghai 201306, China; sl5284sumana@gmail.com

**Keywords:** aquaculture ponds, antibiotics, enrofloxacin, sulfonamides, seasonal variation, LC–MS/MS, antimicrobial resistance, combined use

## Abstract

**Simple Summary:**

Antibiotics are commonly used in aquaculture to prevent disease, but their residues can remain in pond water and affect the environment. In this study, we measured sulfonamide and fluoroquinolone antibiotics in 40 aquaculture ponds around Wuxi, China, during two key farming stages: the summer grow-out period (August) and the autumn harvest (October). Using sensitive LC–MS/MS analysis, we found that antibiotics were widespread. Enrofloxacin, a fluoroquinolone, was dominant in August, occurring in over half the ponds at concentrations up to 2.36 µg/L. By October, sulfonamides such as sulfamethoxazole and sulfadiazine became more common, with one pond showing sulfadiazine levels above 4 µg/L. Statistical analyses confirmed a clear seasonal shift in antibiotic patterns and revealed that multiple sulfonamides were often used together in autumn. These results highlight two key issues: (1) different antibiotics dominate at different farming stages, and (2) combined use of drugs increases potential risks of antimicrobial resistance and ecological harm. To address these concerns, we recommend stricter monitoring and regulation, better farm management to reduce reliance on antibiotics, and promotion of alternative disease-control strategies. This study provides valuable baseline data for improving the sustainable and safe use of antibiotics in aquaculture.

**Abstract:**

Antibiotic use in aquaculture has become widespread to sustain production and control bacterial diseases, but it poses significant ecological and human health risks due to residue accumulation and resistance development. This study investigated the occurrence, dominance, and combined use of sulfonamide and fluoroquinolone antibiotics in freshwater fish aquaculture ponds around Wuxi, China. Here, the term aquaculture refers specifically to the controlled farming of freshwater fish species such as carp and crucian carp in managed pond systems. A total of 80 water samples (collected exclusively from pond waters) were obtained from 40 ponds during the high intensity rearing and harvest stage of fish. Residues of enrofloxacin and sulfonamide antibiotics were analyzed using a validated LC–MS/MS method with detection limits in the low nanogram-per-liter range. Results revealed that antibiotics were ubiquitous in pond waters, with enrofloxacin emerging as the dominant compound in August, reaching concentrations of up to 2.36 µg/L. By October, sulfonamides, particularly sulfamethoxazole and sulfadiazine, became more prevalent, with a maximum sulfadiazine concentration exceeding 4 µg/L. Multivariate analyses demonstrated a clear seasonal shift in antibiotic profiles, while correlation analyses indicated limited combined use in summer but notable co-occurrence of sulfonamides in autumn. These findings underscore that antibiotic application patterns in aquaculture are strongly linked to production stages, with potential consequences for environmental safety, resistance development, and food security. Effective monitoring, stricter regulation, and alternative disease management strategies are urgently required to mitigate risks and promote sustainable aquaculture practices.

## 1. Introduction

Aquaculture has rapidly emerged as one of the most important sectors in global food production, driven by the increasing demand for animal protein and nutrients from seafood [1]. Over the past three decades, the industry has grown at an average annual rate of nearly 6% [2], evolving from small-scale practices to large-scale intensive systems [3,4]. This transformation has been particularly significant in Asia, with China leading global aquaculture production and contributing more than 60% of the total output [4,5]. To sustain such high production levels, aquaculture relies heavily on inputs including formulated feeds, fertilizers, water conditioners, and pharmaceuticals such as antibiotics [6]. Antibiotics are widely applied not only to treat bacterial infections but also to prevent disease outbreaks and promote the growth of cultured species [7,8].

The use of antibiotics in aquaculture has become an essential practice to maintain stock health in high-density systems. For example, salmon farming in Chile and freshwater aquaculture in China both rely on medicated feeds and direct administration of antibiotics into ponds as common disease-control strategies [9]. However, studies indicate that a large proportion of these compounds ranging from 25% to 80% are not metabolized by aquatic organisms and are instead released into the surrounding environment through excretion or as uneaten feed [10,11]. As a result, antibiotics and their metabolites accumulate in pond waters, sediments, and surrounding ecosystems, creating persistent residues that pose ecological and public health risks [12,13].

The scale of antibiotic consumption in aquaculture has become alarming worldwide. Global antibiotic use reached approximately 93 million tons in 2017 and is projected to exceed 236 million tons by 2030, with 5.7% of this total linked to aquaculture production [14]. Although aquaculture accounts for a relatively small fraction of total global antibiotic use compared to livestock, the direct discharge of antibiotics into aquatic environments without adequate treatment amplifies its ecological impact [15,16]. China has been identified as both the world’s largest producer and consumer of antibiotics, with usage in aquaculture exceeding 10,000 tons annually [17]. In 2013 alone, China’s farming of food animals, including fish, accounted for 13,700 tons of antibiotics, representing over 80% of the total national antibiotic discharge [18].

Surveillance studies across China’s major water bodies, including the Yangtze River, Pearl River Delta, Huangpu River, and Taihu Lake, have consistently reported high detection rates of sulfonamides, quinolones, macrolides, and tetracyclines in surface waters and sediments [19,20,21]. Concentrations in aquaculture environments often reach several micrograms per liter in water and milligrams per kilogram in sediments [22,23,24]. For instance, in Jiangsu Province, a major aquaculture hub, average antibiotic concentration in ponds has been reported to be as high as 7174.8 ng/L [25]. Similar findings from aquaculture ponds in the Jianghan Plain revealed peak concentrations of nearly 16,000 ng/L in water and 9000 ng/g in sediment [23]. Such levels not only exceed ecological safety thresholds but also highlight the widespread dominance of antibiotics in aquaculture ecosystems.

Another challenge arises from the combined and overlapping use of multiple antibiotic classes. Surveys in major aquaculture-producing countries show that more than 67 antibiotics are used simultaneously, often without veterinary prescription or awareness of toxicological impacts [26,27]. Oxytetracycline, florfenicol, and sulfonamides remain among the most widely used antibiotics in aquaculture worldwide, primarily employed to combat bacterial pathogens such as *Aeromonas*, *Vibrio*, and *Pseudomonas* species, which commonly infect farmed fish and shrimp. These drugs belong mainly to the sulfonamide, fluoroquinolone, and tetracycline chemical classes, which are routinely administered in fish farms culturing species like carp, tilapia, salmon, and catfish, as well as in shrimp operations across China, Vietnam, Thailand, Chile, and Norway [12,26,27]. Despite growing awareness and regulatory measures, banned compounds such as chloramphenicol and penicillin are still occasionally detected in some Asian aquaculture systems, underscoring gaps in enforcement and monitoring. Such unregulated and overlapping use leads to the so-called “pseudo-persistence” of antibiotics in aquatic environments, where continuous release from uneaten feed, fecal matter, and water exchange exceeds natural degradation through photolysis, hydrolysis, and biodegradation [13,28].

The occurrence and dominance of antibiotics in aquaculture environments have far-reaching ecological and health implications. Firstly, antibiotic residues can directly affect aquatic organisms, particularly phytoplankton communities such as green algae, which are essential for maintaining pond productivity and nutrient cycling [29,30]. Studies indicate that both acute and chronic exposure to antibiotics can inhibit nitrogen assimilation and photosynthesis in algae, thereby reducing primary productivity and disrupting biogeochemical functions [31,32]. This, in turn, threatens ecosystem services such as organic matter oxidation and nutrient recycling, which underpin aquaculture sustainability [33].

Secondly, antibiotics in aquaculture promote the emergence and spread of antibiotic-resistant bacteria (ARB) and antibiotic resistance genes (ARGs) [34]. Low but persistent concentrations in water and sediments create selective pressure for microbial resistance, facilitating horizontal gene transfer among bacterial communities [13,34]. ARGs from aquaculture environments can disseminate into surrounding rivers, lakes, and even drinking water sources, representing one of the most pressing environmental health issues of the 21st century [18]. Additionally, bioaccumulation of antibiotic residues in cultured organisms raises concerns for food safety and human health, as resistant bacteria and residues can enter the food chain [35,36].

Thirdly, the lack of effective wastewater treatment for aquaculture effluents exacerbates the situation. Unlike municipal wastewater, aquaculture discharges often bypass centralized treatment plants, resulting in direct release of antibiotic-laden water into rivers, lakes, and coastal zones [37]. Even where wastewater treatment plants exist, removal efficiencies remain low, often between 36 and 79%, allowing antibiotics to persist across water, sediments, and soils [38]. Consequently, aquaculture has become one of the primary sources of antibiotics entering the aquatic environment globally [1,39].

Despite the mounting evidence of antibiotic occurrence in aquaculture ponds, several critical gaps remain. First, most existing studies have focused on detecting individual antibiotics at single time points, with limited attention to their combined use and interactions across different aquaculture stages [40,41]. Understanding how antibiotic concentrations fluctuate throughout the aquaculture cycle from pond preparation, seeding, and mid-growth to harvest is essential for identifying critical control points.

Second, while sulfonamides, quinolones, tetracyclines, and macrolides have been frequently reported, there is insufficient knowledge on which compounds dominate in specific pond environments and how their dominance shifts spatially and temporally [22,42]. Clarifying the dominant antibiotics in aquaculture ponds can help prioritize monitoring and risk management efforts.

Third, the ecological risks of combined antibiotic use remain poorly characterized. Most risk assessments are conducted for single compounds, ignoring possible additive, synergistic, or antagonistic effects when multiple antibiotics co-occur [43]. Given that multiple antibiotics are commonly applied in aquaculture practices, evaluating their joint ecological risks is crucial.

Finally, regional differences in aquaculture practices and antibiotic inputs complicate efforts to generalize findings. For example, studies in Southern Jiangsu highlight significantly higher antibiotic levels in mid-aquaculture stages compared to early or late stages, while in shrimp farms of southern China, influent water has been identified as the major source of antibiotics [41]. Thus, a systematic approach that considers species differences, pond management, and regional characteristics is needed to provide a more comprehensive understanding of antibiotic pollution in aquaculture.

## 2. Materials and Methods

### 2.1. Study Area and Sampling Design

**Figure 1:**
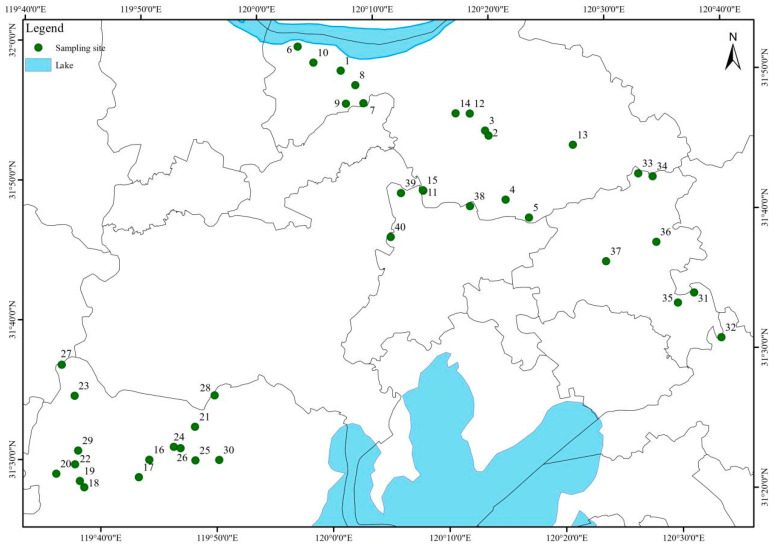
Geographic distribution of sampling sites in the study area. 40 sampling points were established in the whole basin, covering the aquaculture ponds around the river and the Taihu Lake. The sites were strategically selected to capture spatial heterogeneity in environmental conditions and potential antibiotic inputs.

Forty representative ponds were selected to capture spatial variability in farm size, culture practices, and management (Figure 1). These ponds were primarily used for culturing freshwater fish species such as crucian carp (*Carassius auratus*) and grass carp (*Ctenopharyngodon idella*). Surface water composite samples (400 mL) were collected from multiple points within each pond at two production stages: the high-intensity rearing period (August 2024) and the harvest/preparation period (October 2024). In total, 80 water samples (collected from 40 aquaculture pond stations, each sampled twice at two production stages with triplicate sampling per station) were analyzed. Field samples were stored on ice, returned to the laboratory within 24 h, filtered through 0.45 µm membranes, and refrigerated at 4 °C.

### 2.2. Analytical Method (LC–MS/MS)

#### 2.2.1. Chromatographic Conditions

Analyses were performed on an ACQUITY UPLC BEH C18 column (2.1 mm × 150 mm, 1.7 μm, Waters, Milford, MA, USA). The injection volume was 10 μL, with a flow rate of 0.3 mL·min^−1^ and the column temperature maintained at 40 °C. The mobile phase consisted of solvent A (water with 0.1% formic acid) and solvent B (methanol with 0.1% formic acid). A 17 min gradient program was applied as follows: 0 min, 98% A; 0.5 min, 98% A; 8 min, 68% A; 10 min, 30% A; 12 min, 2% A; 14 min, 2% A; 14.5 min, 98% A; and 17 min, 98% A for re-equilibration.

#### 2.2.2. Mass Spectrometric Conditions

Detection was carried out using an electrospray ionization (ESI) source operated in positive mode. The capillary voltage was set at 1.50 kV, and the cone voltage at 48 V. The desolvation gas temperature was 550 °C with a flow rate of 800 L·h^−1^, and the cone gas (sheath gas) flow rate was 50 L·h^−1^. The ion source temperature was maintained at 550 °C. The collision energy was set at 3 eV. In MS/MS mode, data were acquired automatically with a precursor ion scan range of *m*/*z* 100–1000 and a product ion scan range of *m*/*z* 50–800.

### 2.3. Method Validation and Quality Control

The analytical method was developed and validated to ensure reliable detection of the antibiotics at trace levels. Table 1 summarizes key method performance parameters. A set of 15 sulfonamides and 5 quinolone antibiotics commonly used in aquaculture (including sulfamethoxazole, sulfadiazine, sulfathiazole, enrofloxacin, ciprofloxacin, etc.) were chosen as target analytes. Calibration curves were constructed for each compound using at least five concentration levels in the range of ~0.001 to 1 µg/L (covering expected environmental concentrations). All calibration curves showed strong linearity (R^2^ generally >0.99). Limits of detection (LOD) and quantification (LOQ) were determined for each analyte based on signal-to-noise criteria of 3 and 10, respectively. The LOQs achieved were on the order of a few ng/L; for example, typical LOQs ranged from approximately 0.001 to 0.005 µg/L (1–5 ng/L) for both sulfonamides and quinolones (Table 1). Method accuracy and precision were evaluated through recovery experiments: pond water samples were spiked with low concentrations of the target antibiotics (around 0.003–0.03 µg/L for each analyte to mimic environmental levels) and processed through the entire SPE and LC–MS/MS procedure. Average recoveries for the different compounds ranged between about 68% and 81%, with relative standard deviations generally below 10%, indicating satisfactory method accuracy and repeatability. To maintain quality control during sample analysis, each batch of 10 samples included a method blank (to ensure no contamination) and a matrix spike sample. No target antibiotics were detected in blanks, and matrix spike recoveries fell within acceptable ranges (typically 70–110%). These validation results (see Table 1) demonstrate that the LC–MS/MS method is sensitive, precise, and fit for purpose in detecting trace sulfonamides and quinolones in water samples.

### 2.4. Data Analysis

All concentration data for the detected antibiotics were compiled and analyzed to discern patterns of occurrence and co-occurrence. Descriptive statistics (minimum, maximum, mean, median, and detection frequency) were calculated for each antibiotic at the two sampling times (August and October) using Microsoft Excel; the results are presented in Table 2. Graphical visualizations were produced with Origin 2025 software, including bar charts to compare antibiotic concentrations between August and October, and a heatmap of correlation coefficients to examine relationships between compounds. Statistical analysis was carried out in R 4.5.1 (R Foundation for Statistical Computing). A non-metric multidimensional scaling (NMDS) analysis was performed using the vegan package in R to visualize differences in antibiotic composition among samples. The NMDS was based on a Bray–Curtis dissimilarity matrix of square-root-transformed concentration data, allowing us to ordinate the 80 samples (40 sites × 2 times) in two-dimensional space. This analysis helps reveal clustering of samples by sampling period and any outliers. Pearson correlation analysis was also conducted for each pair of antibiotics across the samples within each season (August and October separately) to identify which antibiotics tend to co-occur. The correlation matrices were visualized as color-coded heatmaps for ease of interpretation. All statistical tests (e.g., comparing total antibiotic concentrations between August and October) used a significance level of α = 0.05.

## 3. Results and Discussion

### 3.1. Method Development and Validation Results (Quality Control)

The LC–MS/MS method effectively detected multiple antibiotics in pond water at sub-µg/L levels. All targeted sulfonamide and quinolone compounds were well resolved and exhibited distinct MRM peaks, demonstrating strong selectivity. Validation data confirmed high sensitivity, with most analytes achieving limits of detection (LODs) in the low nanogram per liter range, comparable to previous studies on antibiotics in aquaculture environments [25,42]. Recovery rates of 70–80% were acceptable given the complexity of pond water, aligning with similar multi-residue LC–MS/MS approaches [20]. These findings confirm that the analytical method provided reliable and accurate results, ensuring that observed differences in concentrations reflect true environmental variability rather than analytical artifacts.

### 3.2. Occurrence and Concentrations of Antibiotics in Pond Water (August vs. October)

All 17 target antibiotics (12 sulfonamides and 5 quinolones) were analyzed across 80 water samples. Most compounds were detected in at least some samples, although their detection frequencies and concentrations varied considerably between the August and October sampling events. Table 2 summarizes the detection frequency (percentage of samples in which each antibiotic was detected) and concentration range for both months.

Overall, antibiotics were widespread in pond water. In August, 62.5% of samples contained at least one quinolone, while 35% contained at least one sulfonamide. By October, the pattern shifted: quinolones were present in 57.5% of samples, and sulfonamides were detected in the same proportion (57.5%), reflecting an increased occurrence of sulfonamides later in the production cycle. The total concentration of antibiotics per sample (sum of all detected compounds) ranged from non-detectable levels to several micrograms per liter. Notably, the highest August concentrations were dominated by a fluoroquinolone (enrofloxacin), whereas in October one sulfonamide (sulfadiazine) spiked dramatically in certain samples, as discussed below. Similar temporal variations in antibiotic residues have been observed in other aquaculture regions, underscoring how farming practices and seasonal management influence antibiotic profiles [42].

**Figure 2:**
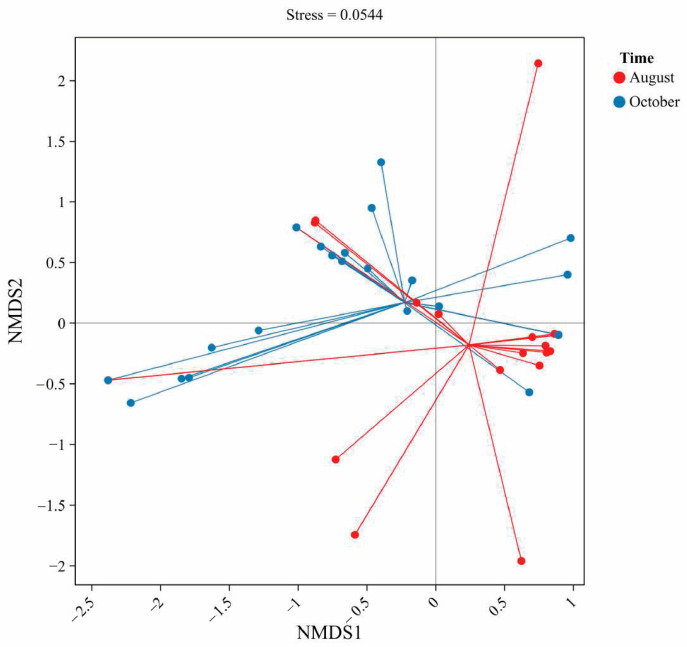
Presents a non-metric multidimensional scaling (NMDS) ordination of antibiotic composition profiles in August and October. The stress value of 0.0544 indicates good ordination reliability. Clustering patterns show partial separation between the two sampling periods, suggesting temporal shifts in antibiotic composition. Longer vectors in August indicate greater heterogeneity compared to October.

From Table 2 and Figure 2, enrofloxacin was the most prominent antibiotic in August. It was detected in 52.5% of water samples, reaching concentrations of ~2.36 µg/L in the most contaminated pond. The average concentration was ~0.64 µg/L, nearly an order of magnitude higher than the next most abundant antibiotic. This pattern reflects the widespread application of enrofloxacin during the peak growing stage, likely due to its effectiveness against common bacterial infections. A similar dominance of fluoroquinolones, particularly enrofloxacin, has been reported in Chinese aquaculture environments [43]. By October, however, enrofloxacin levels had decreased sharply (mean ~0.03 µg/L), although they remained detectable in 57.5% of samples. The decline may result from the cessation of dosing before harvest, natural degradation processes, or dilution by rainfall and water exchange. Farmers may also have intentionally reduced use near harvest to comply with residue regulations in marketable fish [25].

In contrast, sulfonamides showed a complementary trend. During August, they were less frequently detected and at generally lower concentrations. For instance, sulfamethoxazole (SMX) appeared in 22.5% of samples with a mean concentration of ~0.26 µg/L, while sulfadiazine was detected in only 5% of samples (mean ~0.016 µg/L). By October, sulfonamide detection increased substantially. Sulfamethoxazole was present in 45% of samples (double its August frequency) with concentrations up to ~1.48 µg/L, though its mean concentration (~0.21 µg/L) was slightly lower than in August. More strikingly, sulfadiazine rose to 17.5% detection in October, with one pond exhibiting an exceptionally high concentration (~4.58 µg/L; Table 2), which elevated the overall mean to ~0.69 µg/L. This spike likely reflects application of a large dose of sulfadiazine or a sulfa-based formulation during harvest, possibly in response to disease outbreaks or as prophylaxis when handling fish. Other sulfonamides, such as sulfathiazole, sulfamerazine, and sulfamethizole, also showed modest increases in detection rates from August to October for example, sulfamethizole was absent in August but detected in 15% of October samples at low concentrations (Table 2). Collectively, sulfonamides contributed a larger share of the antibiotic burden in October than in August. This seasonal shift may reflect a change in medication strategy: farmers relied on a potent fluoroquinolone (enrofloxacin) during the summer grow-out phase, but by autumn they switched to or supplemented with sulfonamides. Such changes may be driven by concerns about withdrawal times, resistance, or the need to treat different infections late in the cycle.

Several antibiotics were rarely or never detected. For example, lomefloxacin (quinolone) and sulfadoxine (sulfonamide) were absent in both months (0% detection), suggesting either non-use in these ponds or concentrations below detection limits. Pefloxacin was detected in 27.5% of August ponds but absent in October, implying it was applied earlier and later discontinued. Other compounds, such as ofloxacin and minor sulfonamides (sulfaquinoxaline, sulfafurazole), were found only sporadically in one or two August samples. These sporadic detections likely represent isolated use or cross-contamination. The rarity of such compounds suggests that actual antibiotic practices may be narrower than the target analyte list, dominated primarily by enrofloxacin and a few sulfonamides (especially sulfamethoxazole and sulfadiazine).

Forty sampling points were established across the study basin, covering aquaculture ponds distributed around the river network and the Taihu Lake region. These ponds were primarily used for culturing freshwater fish species such as crucian carp (*Carassius auratus*) and grass carp (*Ctenopharyngodon idella*). The locations were strategically selected to represent spatial heterogeneity in environmental conditions and potential sources of antibiotic inputs. Although there were no nearby chemical or pharmaceutical industries or major medical facilities, the surrounding landscape consisted largely of agricultural land and fish farms. This indicates that any antibiotic residues detected were more likely derived from aquaculture activities and diffuse agricultural runoff rather than direct industrial discharges.

Overall, the antibiotic residue profiles in this study align with findings from other aquaculture regions, where a small number of antibiotics dominate residue patterns. Sulfonamides and fluoroquinolones have consistently been reported as the primary antibiotic classes in aquaculture systems [27]. Our results reinforce that enrofloxacin (fluoroquinolone) and sulfamethoxazole/sulfadiazine (sulfonamides) are critical compounds of concern due to their high prevalence and concentrations, underscoring the need for targeted monitoring and regulatory attention.

### 3.3. Dominant Antibiotics and Seasonal Variation

Enrofloxacin emerged as the dominant antibiotic during August, contributing the largest share of total antibiotic residues. In October, sulfonamides such as sulfamethoxazole and sulfadiazine gained prominence, indicating a seasonal shift. Such patterns likely result from differences in disease pressure, management practices, and withdrawal time considerations before harvest [5,31]. Environmental fate may also contribute to these trends: fluoroquinolones such as enrofloxacin tend to adsorb to sediments and degrade under sunlight, while sulfonamides are more water-soluble and persistent [28]. These findings confirm that aquaculture management cycles strongly influence antibiotic profiles across production seasons.

### 3.4. Co-Occurrence and Combined Use of Antibiotics (Correlation Analysis)

**Figure 3:**
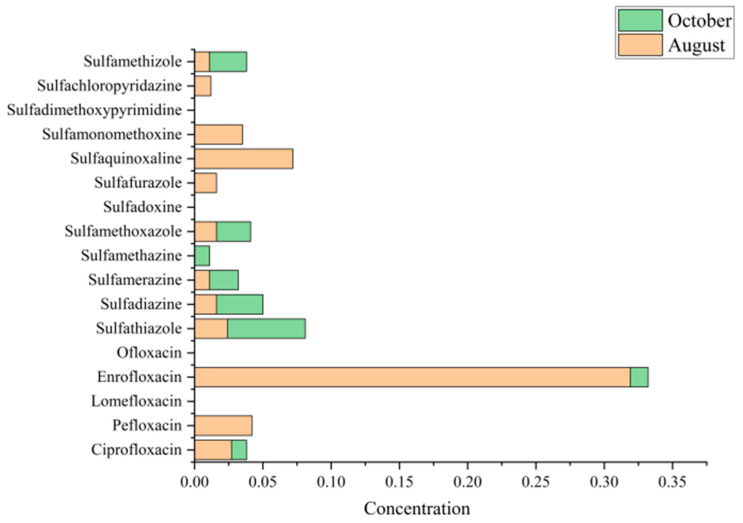
Seasonal variation in median antibiotic concentration in aquaculture ponds samples collected in August and October. Concentrations of sulfonamides and fluoroquinolones are shown. Among the detected compounds, enrofloxacin exhibited the highest concentration in both seasons, with particularly elevated levels in August, whereas other antibiotics such as sulfathiazole and sulfamethoxazole showed relatively higher values in October.

We investigated the co-occurrence patterns of the antibiotics to see if certain compounds tend to appear together in the same water samples, which can indicate combined use or common sources. Figure 3 presents heatmaps of Pearson correlation coefficients between each pair of antibiotics, calculated separately for the August and October sample sets. In these matrices, a warmer color (e.g., red) signifies a strong positive correlation (i.e., the two antibiotics tend to have high concentrations in the same samples), whereas cooler colors (blue) indicate negative or no correlation.

From Figure 3, it is apparent that the co-occurrence patterns differed markedly between the two sampling periods. In August, most pairwise correlations between different antibiotics were weak or insignificant (cells are mostly pale or light-colored, indicating r values near zero). This implies that during the peak growth period, farmers might have predominantly used one primary antibiotic (enrofloxacin) across many ponds, with only sporadic use of others leading to little overlap in the occurrence of multiple antibiotics in the same water sample. Similar observations have been made in other aquaculture studies where enrofloxacin is commonly applied as the first-line antibiotic [31].

In October, the correlation heatmap displays a different scenario. There are several red-highlighted cells forming a cluster, especially among the sulfonamides, indicating strong positive correlations in that month. For example, sulfamethoxazole concentrations were strongly correlated with sulfamethizole and sulfadiazine in October (as shown by high r values approaching 0.8–0.9 in the heatmap). This suggests that in the ponds where sulfamethoxazole was found at elevated levels, sulfadiazine and sulfamethizole (and possibly other sulfonamides) were often present as well. Such a pattern implies combined use of multiple sulfonamides (or a broad-spectrum sulfa drug formulation) in certain ponds during the harvest period. Similar combined applications of antibiotics in aquaculture have been reported in other regions, raising concerns for multi-drug resistance [12,27].

The August data did not show such clustering of high correlations, indicating that broad simultaneous use of multiple antibiotics was not common in the summer samples. By contrast, the October data revealed concurrent sulfonamide use, suggesting that farmers may turn to mixtures or sequential treatments during later production stages. This combined presence of antibiotics highlights the potential for additive or synergistic effects and increased risks of antibiotic resistance development [34].

### 3.5. Differences in Antibiotic Profiles Between August and October (NMDS Analysis)

**Figure 4:**
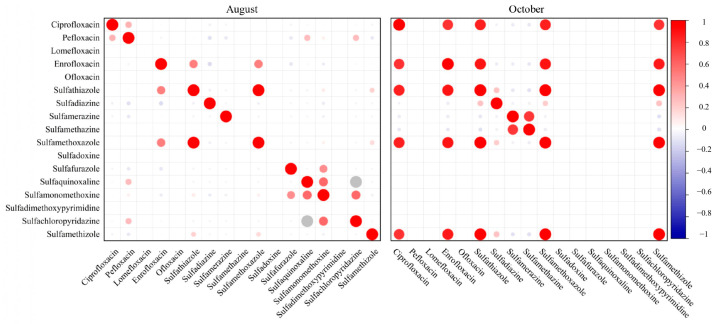
Heatmap of antibiotics in aquaculture ponds during August and October.Circle size indicates correlation strength, and circle color represents the sign of the correlation (red = positive, blue = negative). Strong positive correlations were observed among multiple sulfonamides and between fluoroquinolones in both months, but the co-occurrence patterns varied between seasons, suggesting temporal differences in antibiotic usage or degradation dynamics.

To visualize the overall differences in antibiotic residue profiles between the two seasons, a non-metric multidimensional scaling (NMDS) analysis was performed on the sample data. The NMDS ordination (based on Bray–Curtis similarity of antibiotic concentrations) provides a two-dimensional map where each point represents a water sample, and the distance between points reflects the similarity of their antibiotic composition. Figure 4 shows the NMDS plot for all 80 samples, with samples coded by sampling month.

The NMDS results underscore a pronounced seasonal shift in the antibiotic contaminant profile of the pond waters. As seen in Figure 4, the red points (August samples) cluster towards one side of the plot, while the blue points (October samples) cluster towards the opposite side, with relatively little overlap. Statistically, this separation suggests that within-group similarity (samples from the same month) is higher than between-group similarity, confirming that August and October samples form distinct groups in terms of antibiotic composition. Such seasonal differences in antibiotic occurrence have also been observed in other aquaculture systems, reflecting changes in usage across farming cycles [42].

Several observations from the NMDS support the earlier univariate analyses. The August cluster is tighter and more isolated, which likely reflects the dominance of enrofloxacin in those samples many August ponds had similar profiles (high enrofloxacin, low others). This pattern aligns with reports that fluoroquinolones often dominate aquaculture residues during peak growth periods [42]. The October samples, while also forming a cluster, show a wider spread. This may be due to greater variability among ponds in autumn, driven by differential sulfonamide use across farms. Some ponds remained relatively clean by harvest, whereas others showed recent dosing events with unique antibiotic signatures. Similar late-stage variability has been documented in other Chinese aquaculture regions [23].

The separation along the NMDS axis suggests that August samples were mainly influenced by fluoroquinolone residues, while October samples reflected sulfonamide inputs. This seasonal effect aligns with management practices, as farmers adjust medications between the growth and harvest stages. Overall, these results highlight the importance of considering seasonal dynamics when assessing antibiotic risks in aquaculture ponds.

## 4. Conclusions

This study examined the occurrence and seasonal variation in sulfonamide and fluoroquinolone antibiotics in aquaculture ponds around Wuxi, China, using LC–MS/MS. Residues were widespread, with clear seasonal differences. During the August grow-out phase, enrofloxacin dominated, detected in over half the samples at concentrations up to several µg/L. By the October harvest period, sulfonamides (notably sulfamethoxazole and sulfadiazine) became more prevalent, while enrofloxacin levels declined. Multivariate NMDS analysis confirmed a temporal separation in antibiotic profiles, reflecting shifts in farming practices and environmental factors. Correlation analysis indicated limited co-use of antibiotics in summer, but stronger co-occurrence of sulfonamides in some autumn samples.

These findings suggest monitoring priorities should differ by season: enrofloxacin during intensive growth, and broader coverage including sulfonamides at harvest. The seasonal shift underscores the need for prudent antibiotic use throughout the cycle. Combined detections in harvest samples raise concerns about additive ecological risks and antimicrobial resistance, even when individual residues meet safety standards. For example, a sulfadiazine spike exceeding 4 µg/L highlights the risk of misuse or overuse.

To mitigate risks, aquaculture should adopt improved management practices, including regular health monitoring to reduce reliance on antibiotics, and strict adherence to withdrawal times when treatments are necessary. Preference should be given to drugs with shorter environmental persistence. Regulatory authorities should strengthen oversight and implement long-term monitoring programs across water, sediments, and biota to track trends, identify hotspots, and evaluate policy effectiveness.

In conclusion, this work provides a baseline of antibiotic pollution in Wuxi’s aquaculture ponds and emphasizes seasonally informed management. Identifying dominant compounds and their usage patterns offers guidance for targeted interventions, helping to safeguard both aquaculture sustainability and environmental health.

## Figures and Tables

**Table 1:** Liquid-mass parameters.

LC–MS/MS Conditions		Chromatographic Conditions	
Parameter	Setting	Parameter	Setting
Instrument	Waters Xevo TQD	Column	ACQUITY UPLC BEH C18 (2.1 mm × 150 mm, 1.7 μm)
Ionization Mode	ESI (positive mode)	Injection Volume	10 μL
Capillary (kV)	1.5	Flow Rate	0.3 mL·min^−1^
Cone (V)	48	Column Temperature	40 °C
Desolvation Temp (°C)	550	Mobile Phase A	Water + 0.1% formic acid (FA)
Desolvation (L/h)	800	Mobile Phase B	Methanol + 0.1% formic acid (FA)
Cone (L/h)	50	Gradient Program	0.0 min: 98% A → 0.5 min: 98% A → 8.0 min: 68% A → 10.0 min: 30% A → 12.0 min: 2% A → 14.0 min: 2% A → 14.5 min: 98% A → 17.0 min: 98% A
Source Temp (°C)	550		
Collision Energy (eV)	3		
LM Resolution 1	9.7		
HM Resolution 1	15	**Sample and Analyte Information**	
Ion Energy 1	0.1	Parameter	Setting
LM Resolution 2	8.7	Analytes	Sulfonamides, Quinolones
HM Resolution 2	15	Sample Volume	400 mL water sample
Ion Energy 2	0.4	Spiked Concentration	0.003 μg/L–0.03 μg/L
Precursor ion scan	*m*/*z* 100–1000	Recovery Rate	68.5–81.0%
Product ion scan	*m*/*z* 50–800	LOQ	0.001–0.005 μg/L

**Table 2:** Antibiotic characteristics in August and October.

Time	August					October				
Antibiotic	Detection Rate	Min (μg/L)	Max (μg/L)	Median (μg/L)	Mean (μg/L)	Detection Rate	Min (μg/L)	Max (μg/L)	Median (μg/L)	Mean (μg/L)
Ciprofloxacin	17.5%	0.011	0.456	0.027	0.091	7.5%	0.005	0.016	0.011	0.011
Pefloxacin	27.5%	0.018	0.106	0.042	0.044	/				
Lomefloxacin	0.0%					0.0%				
Enrofloxacin	52.5%	0.203	2.360	0.319	0.645	57.5%	0.008	0.257	0.013	0.034
Ofloxacin	/					/				
**Quinolones**	**62.5%**	**0.042**	**2.404**	**0.341**	**0.586**	**57.5%**	**0.008**	**0.273**	**0.013**	**0.035**
Sulfathiazole	10.0%	0.012	0.103	0.024	0.041	12.5%	0.019	0.142	0.057	0.068
Sulfadiazine	5.0%	0.010	0.021	0.016	0.016	17.5%	0.013	4.575	0.034	0.689
Sulfamerazine	2.5%	0.011	0.011	0.011	0.011	10.0%	0.012	0.041	0.021	0.023
Sulfamethazine	0.0%					10.0%	0.006	0.014	0.011	0.011
Sulfamethoxazole	22.5%	0.011	1.526	0.016	0.260	45.0%	0.009	1.475	0.025	0.213
Sulfadoxine	/					/				
Sulfafurazole	2.5%	0.016	0.016	0.016	0.016	/				
Sulfaquinoxaline	2.5%	0.072	0.072	0.072	0.072	/				
Sulfamonomethoxine	12.5%	0.010	0.044	0.035	0.030	/				
Sulfadimethoxypyrimidine	/					/				
Sulfachloropyridazine	2.5%	0.012	0.012	0.012	0.012	/				
Sulfamethizole	2.5%	0.011	0.011	0.011	0.011	15.0%	0.010	0.061	0.027	0.028
**Sulfonamides**	**35.0%**	**0.010**	**1.629**	**0.017**	**0.249**	**57.5%**	**0.006**	**5.104**	**0.034**	**0.607**

## Data Availability

The raw data supporting the conclusions of this article will be made available by the authors on request. the data generated and used in this study belong to the Central Public interest Scientific Institution Basal Research Fund, Freshwater Fisheries Research Center CAFS (NO 2025JBFZ04), Central Public-interest Scientific Institution Basal Research Fund, CAFS (NO 2025XT0404) and China Agricultural Research System (CARS-46), Wuxi Science and Technology Development Fund (K20231029). According to the data management and confidentiality policies of this program, all project data must undergo a period of internal management and strict desensitization and security review before public release to ensure compliance with relevant national regulations. Therefore, the data have not yet been deposited in a public repository. We sincerely affirm that the relevant data can be used to verify the results of this study. Upon receipt of reasonable research requests, we will provide appropriately processed data to interested researchers in accordance with the project’s management rules and confidentiality requirements, and in a manner consistent with national policies and intellectual property protections. We appreciate your understanding and welcome any further inquiries you may have.
